# Benzothiadiazole enhances ascorbate recycling and polyphenols accumulation in blueberry in a cultivar-dependent manner

**DOI:** 10.3389/fpls.2022.1032133

**Published:** 2022-12-09

**Authors:** Giacomo Cocetta, Beatrice Cavenago, Roberta Bulgari, Anna Spinardi

**Affiliations:** ^1^ Department of Agricultural and Environmental Sciences, Università Degli Studi di Milano, Milano, Italy; ^2^ Department of Agricultural, Forest, and Food Sciences (DISAFA), Vegetable Crops and Medicinal and Aromatic Plants VEGMAP, University of Torino, Torino, Italy

**Keywords:** *Vaccinium corymbosum*, phenols, anthocyanin profile, antioxidants, fruit quality, plant disease resistance inducers

## Abstract

Benzothiadiazole (BTH) is a functional analogue of salicylic acid able to induce systemic acquired resistance in many horticultural crops. The aim of the work was to investigate how BTH may affect i) fruit quality, ii) ascorbic acid (AsA) oxidation and recycling metabolism and iii) phenolic compounds accumulation, during development and ripening of berries from the two selected cultivars. Blueberry (*Vaccinium corymbosum* L.) plants (cv ‘Brigitta’ and ‘Duke’) were treated with 0.118 mM BTH every two weeks during ripening, then all fruits of each plant were harvested and divided in four developmental stages. Results indicated that BTH had no marked effects on fruit quality parameters. During the first developmental stage, BTH negatively affected dry matter in both cv, while soluble solids and AsA content were affected in ‘Duke’. In fully ripe berries, BTH reduced dry matter in ‘Duke’ and enhanced soluble solids content in ‘Brigitta’, while diminishing titratable acidity. AsA content was positively affected by BTH in ‘Duke’, but not in ‘Brigitta’. The effect of BTH on the enzymes involved in AsA recycling was recorded in berries at the third (fruit more than half pigmented) and fourth developmental stages. After treatment, in both cv ascorbate peroxidase (APX) activity increased in fully ripe berries, while monodehydroascorbate reductase (MDHAR) activity was stimulated at the third ripening stage. Conversely, the activities of dehydroascorbate reductase (DHAR) and glutathione reductase (GR) were enhanced only in ‘Brigitta’ and in ‘Duke’, respectively. BTH stimulated total polyphenols, flavonoid and anthocyanin accumulation in ‘Brigitta’ and in ‘Duke’ at the third and fourth ripening stages. In fully ripe berries, BTH enhanced the accumulation of delphinidins, cyanidins, petunidins and peonidins in ‘Brigitta’, while in ‘Duke’ it increased all classes of anthocyanidins, including malvidin. On the contrary, the relative proportion of the individual anthocyanins was only slightly affected by BTH treatment, mainly regarding delphinidin and malvidin at the third and fourth stage of ripening of ‘Duke’ and ‘Brigitta’, respectively. These results show that preharvest BTH application can positively impact on fruit bioactive compounds levels, affecting AsA recycling and content and increasing polyphenols accumulation in fruit, but partly depending on cv and ripening stage.

## 1 Introduction

**Figure 1:**
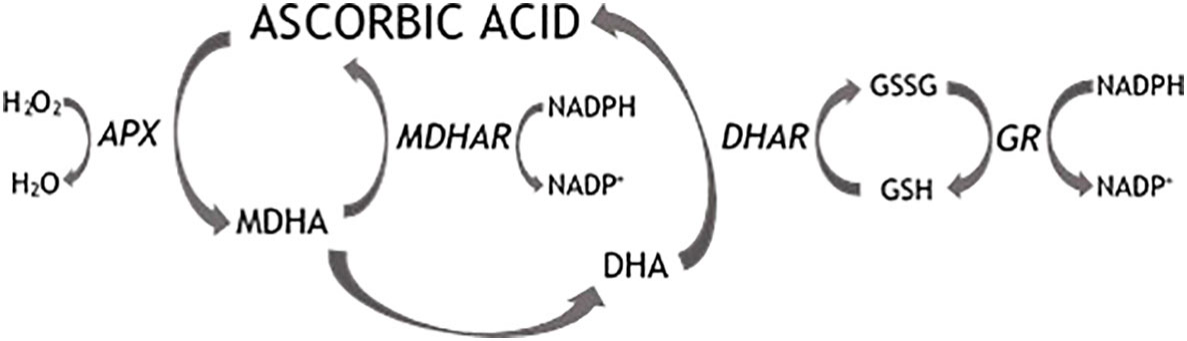
Ascorbic acid recycling pathways. APX, ascorbate peroxidase; MDHAR, monodehydroascorbate reductase; DHAR, dehydroascorbate reductase; GR, glutathione reductase; MDHA, monodehydroascorbate; DHA, dehydroascorbate.

The Fruits consumption plays an important role as a health-promoting factor and is mainly correlated with the antioxidant activity of several compounds which are largely present in plant products. Blueberry (*Vaccinium corymbosum* L.) is one of the fruits with the highest antioxidant potential, due to the high polyphenols concentration and to the moderate presence of ascorbic acid (AsA). AsA is an essential constituent of the human diet, since humans are unable to synthesize it. AsA is one of the most important free radical scavengers in plants, animals and humans. In mammals, AsA plays a significant role in counteracting various oxidative stress-related diseases such as cardiovascular diseases, cancers, aging, as well as stimulating the immune system (Lee, 2019). Furthermore, as the most efficacious and least toxic antioxidant, AsA interacts in plants (enzymatically and non-enzymatically) with damaging oxygen radicals and their derivatives ROS (reactive oxygen species), originated as byproducts of normal cellular metabolism in chloroplasts, mitochondria and peroxisomes (Smirnoff, 2011). The toxicity of ROS is linked to their ability to trigger radical cascade reactions which can cause lipid peroxidation, protein and DNA damage and lead finally to cell death (Blokhina et al., 2003). Oxidative stress is a condition originated by the intracellular accumulation of toxic levels of ROS through saturation of the antioxidant enzymatic and non-enzymatic defence mechanisms. AsA is oxidized by enzymatic or non-enzymatic reactions. Ascorbate peroxidase (APX) mediates the scavenging of hydrogen peroxide to water with the simultaneous oxidation of AsA with a high specificity (**Figure 1**). Ascorbate oxidation leads to the intermediate monodehydroascorbate (MDHA) radical which normally has a short life span and, if not rapidly reduced by monodehydroascorbate reductase (MDHAR), spontaneously dismutates into ascorbate and the labile dehydroascorbate (DHA). DHA is reduced to ascorbate by the action of dehydroascorbate reductase (DHAR), using glutathione (GSH) as the reducing substrate. DHA can undergo irreversible hydrolysis to form 2,3-diketo-L-gulonic acid with consequent loss of biological activity (Parsons et al., 2011) or is directly catabolized to a number of breakdown products including oxalate and tartrate (Loewus, 2012). Due to the interaction with ROS, AsA is also involved in the modulation of several processes, including cell division, lignification and in incompatible plant-pathogen interactions (Conklin, 2001; Bilska et al., 2019). When incompatible plant-pathogen interactions occur, the recognition of an invading pathogen triggers an ‘oxidative burst’ and a coordinated defense response, mediated by ROS.

Blueberries are a rich source of phenolic compounds, a broad class of secondary metabolites synthesized in the phenylpropanoid pathway and acting as antioxidants (Shi et al., 2017; Maya-Cano et al., 2021). Recent studies have demonstrated that the presence of polyphenols in food is particularly important for their oxidative stability and anti-microbial protection (Smeriglio et al., 2016; Durazzo et al., 2019; Garzón et al., 2020). In general, the role of secondary metabolites in plant defense mechanisms is related to their particular chemical and physical characteristics that make them poisonous or repellent. For example, during fruit ripening several changes in the phenolic compounds pool take place determining a decrease in astringency and increase in the visual appeal with most color deriving from anthocyanins (Günther et al., 2020). The levels of flavonoids, proanthocyanins, and hydroxycinnamic acids are higher in young blueberries and tend to diminish at the latest stages of fruit development, while anthocyanin biosynthesis starts at the onset of ripening and reaches its maximum in fully ripe fruit. Their accumulation begins at the onset of ripening, and they represent the major flavonoids in the ripe berry (Castrejón et al., 2008; Zifkin et al., 2012; Karppinen et al., 2016).

Phenylalanine ammonia lyase (PAL), the key enzyme of the phenolic compounds biosynthesis, is influenced by many environmental factors, including light (through its effects on phytochrome), temperature, concentration of nutrients (Sanchez-Ballesta et al., 2000). Many phenolic compounds possess antimicrobial activity that can effectively combat fungal, bacterial or viral infections (Vermerris and Nicholson, 2008; Othman et al., 2019; Walsh et al., 2019; Parham et al., 2020). The inducible defence mechanism that provides a long-lasting, systemic resistance against broad spectrum of pathogens is called systemic acquired resistance (SAR). The resistance process of SAR is mediated by the increase of endogenous salicylic acid (SA). Plants possess two pathways to synthesize SA (Lefevere et al., 2020). SA was originally proposed to be synthesized from Phenylalanine downstream the biosynthetic pathway started by PAL (Dempsey et al., 2011), but Wildermuth et al. (2001) reported that pathogen-induced SA is synthesized through the isochorismate pathway. It is activated at the site of infection as well as in distant uninfected tissues. Plants treatment with biotic and abiotic agents (e.g. plant pathogens, nonpathogens, plant extracts, cell wall fragments and synthetic chemicals) can trigger SAR and lead to the induction of resistance to subsequent attack from pathogens (Oliveira et al., 2016). Frequently, SAR depends on the early increase of SA, which induces a specific set of defense genes. These genes include those coding for pathogenesis-related-proteins (PR), that may contribute to the resistance *via* hydrolysis of the cell wall of pathogen, and key enzymes of secondary metabolic pathways, thus improving phytoalexin synthesis and, subsequently, plant resistance. However, some studies report that induced resistance by pathogens or synthetic SAR inducers affect plant metabolism broadly, exerting effects on both primary and secondary metabolism. Defence mechanisms may additionally be induced by physical or chemical triggers such as application of elicitors. These may stimulate the production of various compounds, particularly flavonoids, lignin and hydroxyproline-rich glycoproteins (Gil-Muñoz et al., 2017). Among the chemical synthetic activators (inducers) of plant disease resistance, the benzothiadiazole (BTH) acibenzolar-S-methyl, is a well-studied functional analogue of SA (Bektas and Eulgem, 2015; Sillero et al., 2012; Yi et al., 2012) which is able to induce SAR in monocots and in many horticultural crops and fruit trees.

The effectiveness of BTH has been observed on strawberries against powdery mildew infection (Hukkanen et al., 2007), tobacco against blue mold (Perez et al., 2003), pea against *Uromyces pisi* (Barilli et al., 2010), and tomato against Cucumber mosaic virus (Anfoka, 2000). The effect of BTH treatments on antioxidative metabolism has also been described. For example, it increased peroxidase (POX), superoxide dismutase (SOD) and ascorbic acid content in peach fruit (Liu et al., 2005). Treatment of soybean cells resulted in increased glutathione reductase (GR), MDHAR and glutathione-S-transferase (GST) activities, as well as higher AsA and GSH content (Knörzer and Böger, 1999). However, BTH was found to inactivate catalase and APX (Wendehenne et al., 1998), the two major H_2_O_2_ scavenger enzymes; accordingly, an increase in H_2_O_2_ occurred in treated tissues, suggesting that changes in H_2_O_2_ levels or in cellular redox state may be involved in BTH/SA-mediated activation of certain defence responses (Wendehenne et al., 1998; Zhu et al., 2016). BTH treatments were also demonstrated to be effective on phenolic metabolism, in fact BTH induced enzyme activities related to anthocyanin metabolism in strawberry fruit after harvest (Cao et al., 2011), enhanced resveratrol, anthocyanin and proanthocyanidin accumulation and resistance to *Botrytis cinerea* in grape (Iriti et al., 2004; Iriti et al., 2005). BTH has been used to prevent fungal infection as an alternative, or complement to fungicide treatments because of its low toxicological risk and rapid degradation in plant tissues (Buonaurio et al., 2002; Johnson et al., 2016)

The aim of this study was to investigate the effects of preharvest spray treatments with BTH on health-promoting properties of two different blueberry cultivars at different stages of ripening, focusing on the increase in antioxidants (enzymatic and non-enzymatic) in the fruit. There is a lack of information on preharvest BTH treatments generally on fruit trees and especially on blueberry. The only research on this fruit has been performed in postharvest studies (Ge et al., 2019). In field or greenhouse, BTH spray treatments have been used on Yali pear (Cao and Jiang, 2006), on strawberry (Hukkanen et al., 2007), on grape (Iriti et al., 2004; Gil-Muñoz et al., 2017), and on apple fruit and leaves (Brisset et al., 2000; Roux et al., 2006). For this reason, the analysis of response of blueberry to BTH, effective in promoting the accumulation of antioxidants like AsA and phenolic compounds, is particularly interesting. The content of AsA was determined. AsA is active in maintaining cellular redox state during stress conditions that increase ROS production. Moreover, the specific activities of the enzymes involved in AsA recycling route were measured. The effect of treatment on phenolic compounds was also assessed, by measuring the levels of total polyphenols, total flavonoids and total anthocyanins, as well as changes in some quality parameters, including total titratable acidity and soluble sugars content

## 2 Material and methods

### 2.1 Chemicals

Standards of cyanidin (Cy)-, delphinidin (D)-, petunidin (Pt)-, peonidin (Pe)-, malvidin (Mv)- and their 3-O-glucoside (glc), Cy-, Pt-, Pe-, Mv-3-O-galactoside (gal) and Cy-arabinoside (Cy-ara) were purchased from Polyphenols Laboratory (Sandnes, Norway). Potassium chloride, hydrochloric acid, methanol, acetonitrile, phosphoric and trifluoroacetic acid (TFA) were from Merck (Darmstadt, Germany). Water was from Milli-Q apparatus (Millipore, Milford, MA).

### 2.2 Plant material

Blueberry (*Vaccinium corymbosum*, L.) were grown in Berbenno (46,168° N; 9,746° E), in Northern Lombardy (Italy) at 650 meters on sea level. The soil of the orchard was characterized by sandy texture class (sand >71%), acidic pH (4.98), and high organic matter content (3.15%), optimal for blueberry growth. Conventional farming practices and micro-irrigation were carried out in the field. Berry samples were collected from 7-year-old plants of two different cultivars, ‘Brigitta’ and ‘Duke’, 24 hours after spray treatments with 0.118 mM BTH (trade name: Bion^®^, Syngenta). Treatments were performed every two weeks starting from the appearance of fruit at color break throughout the growing season (in total 3 applications). ‘Duke’ is an early ripening variety, harvested in Northern Italy in the middle of June—first week of July, whereas ‘Brigitta’, a mid-season variety, ripens in the middle of July-first week of August. ‘Duke’ berries were harvested in the third week of June, and ‘Brigitta’ berries were sampled in the third week of July.

**Figure 2:**
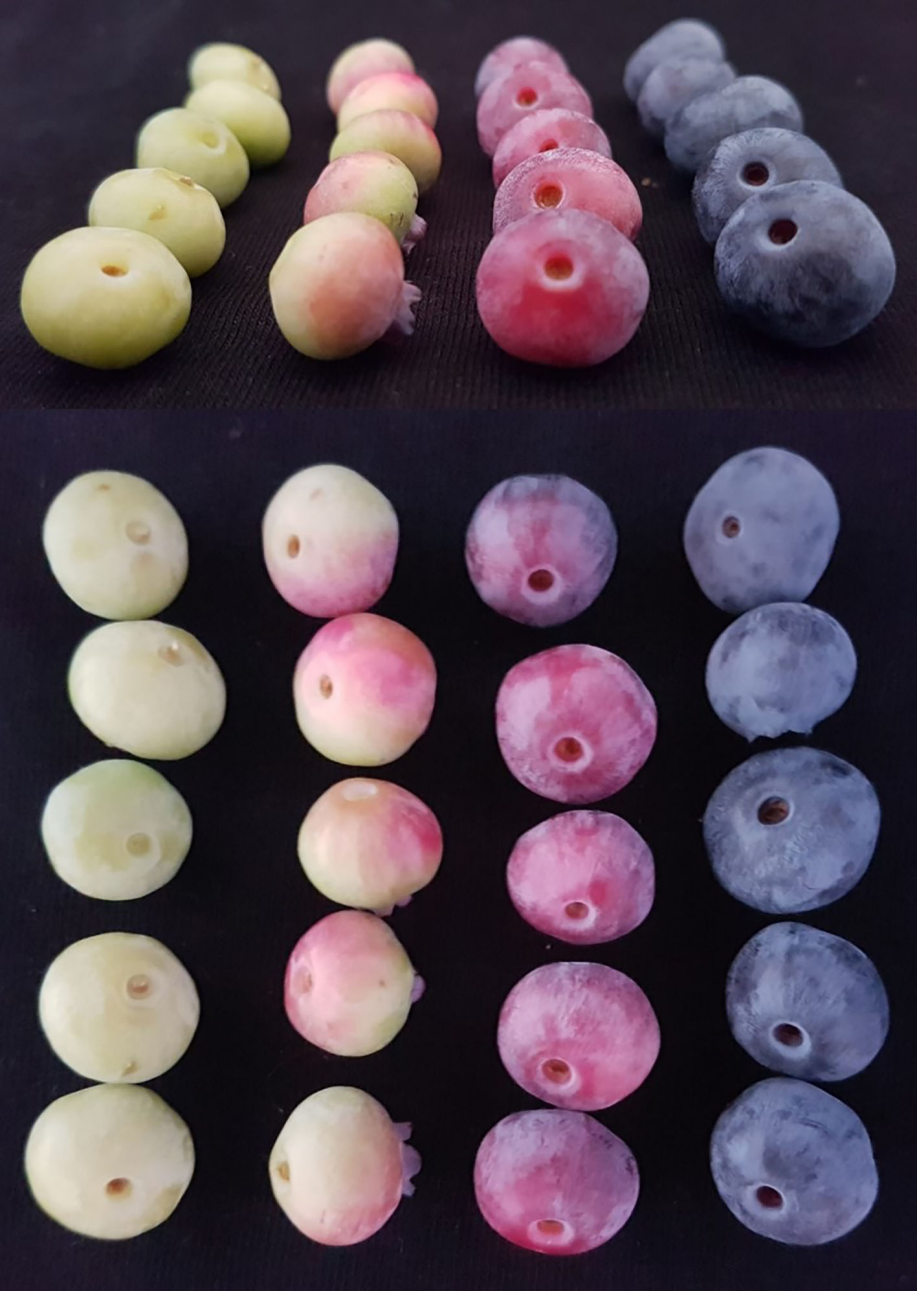
Different ripening stages of blueberry fruit (from left, stage 1: mature green, stage 2: less than 50% pigmented, stage 3: 50% to completely pigmented except the stem end, stage 4: fully ripe).

Fruits from plants treated with water were also collected as a control. All BTH or water treated berries of each bush were picked and, as blueberries do not ripen simultaneously, sorted into four homogenous classes corresponding to different stages of ripening: mature green, fully expanded (class 1), less than 50% pigmented (class 2), 50% to completely pigmented except the stem-end (class 3), and fully ripe (class 4). After BTH application, six treated plants and six control plants were randomly selected for the sampling. Plants were uniform in vegetative development and size. Consequently, the pool of berries harvested from the same plant at the assigned stage of ripening represented one biological replicate for each cultivar, and the total number of samples was 96 (4 ripening stages per 6 individual plants per 2 cultivars per 2 treatments) (**Figure 2**).

Berries of every different ripening stage were then weighed, placed in plastic bags and stored at -80°C until laboratory analysis.

### 2.3 Total titratable acidity determination

The total titratable acidity (TTA) was determined on 5 g of berries following homogenization of the fruits with an equal weight of water for 5 min. The homogenate was titrated to pH 8.3 with 0.1 N NaOH with a Crison automatic titrator (Crison Instruments A. G., Baar, Switzerland), and TTA was calculated and expressed as milliequivalents per 100 grams of fresh weight (FW).

### 2.4 Analysis of water content

To measure the water content of the samples, the intact berries were freeze-dried and weighted before and after the procedure, until no more decrease of weight was recorded.

### 2.5 Total soluble solids determination

Total soluble solids, expressed as °Brix, were determined by a hand refractometer (Atago mod., N1, Tokyo, Japan) on juice obtained from squeezing 5 g of berries.

### 2.6 Determination of AsA content

For ascorbate analysis, 7.5 g of blueberries were homogenized in a mortar with 10 mL of cold 6% (w/v) metaphosphoric acid and centrifuged at 10,000 × *g* at 4°C. The supernatant was transferred into a 25-mL volumetric flask at 4°C. The pellet obtained by centrifugation was washed with 8 mL of cold metaphosphoric acid solution and centrifuged. The supernatants were combined and cold 6% metaphosphoric acid was added to a final volume of 25 mL. After filtration through 0.2-μm nylon filter, a 10 μL sample aliquot was injected onto an Inertsil ODS-3 (5 μm; 4.6mm×250mm) GL Science column at 20°C attached to a Series 200 LC pump (PerkinElmer, Norwalk, CT, USA). The column was eluted with 0.02 M-orthophosphoric acid at a flow rate of 0.7 mL/min in isocratic elution and ascorbic acid was monitored at 254 nm with a UV-975 intelligent UV–vis detector (Jasco model 7800, Tokyo, Japan). Peaks were converted to concentrations by using the dilution of stock ascorbic acid to construct a standard curve. Chromatographic data were stored and processed with a Perkin Elmer TotalChrom 6.3 data processor (PerkinElmer, Norwalk, CT, USA) (Sinelli et al., 2008).

### 2.7 Analysis of enzyme activities

Samples of berries (5 g) from stages 3 and 4 were homogenized in a mortar at 4°C with 10 mL of 100 mM potassium phosphate buffer (pH 7.8) containing 1 mM EDTA, 25% glycerol (w/v), 0.25% Triton X-100 (w/v) and 1 g of polyvinylpolypyrrolidone (PVPP). Just before the extraction, 2 mM β-mercaptoethanol, 1 mM phenylmethylsulfonyl fluoride (dissolved in dimethyl sulfoxide), and 1 mM sodium ascorbate were added to the buffer. The homogenate was filtered through two layers of cheesecloth and centrifuged at 20,000 *g* at 4°C for 30 min to separate insoluble material. The supernatant was then stored at -80°C until used for analyses of enzyme activities.

Protein content was determined according to Bradford (1976) using bovine serum albumin (BSA) as a standard. The enzyme activities were measured with a Cary 50 Bio spectrophotometer (UV-Visible) (Varian Australia Ptv Ltd., Victoria, Australia).

### 2.8 APX (EC 1.11.1.11) activity assay

APX activity was assayed by measuring the decrease in the ascorbate concentration at 290 nm (extinction coefficient: 2.8 mM^-1^ cm^-1^) according to Nakano and Asada (1981) with slight modifications. The assay mixture consisted of 50 mM potassium phosphate buffer (pH 7.0) supplemented with 0.1 mM EDTA and 0.5 mM sodium ascorbate. The reaction was triggered by adding 0.1 mM H_2_O_2_.

### 2.9 MDHAR (EC 1.6.5.4) activity assay

The activity of MDHAR was measured by observing the decrease in absorbance at 340 nm due to oxidation of NADH (extinction coefficient: 6.22 mM^-1^ cm^-1^) according to Arrigoni et al., (1981) with slight modifications. The MDHA was generated by the ascorbate/ascorbate oxidase complex. The assay mixture consisted of 50 mM Tris-HCl buffer (pH 7.6) supplemented with 2.5 mM sodium ascorbate and 0.1 mM NADH. The reaction was triggered by adding 0.14 U of ascorbate oxidase.

### 2.10 DHAR (EC 1.8.5.1) activity assay

DHAR activity was measured by observing the increase in absorbance at 265 nm due to the formation of ascorbate (extinction coefficient: 14 mM^-1^ cm^-1^). The reaction mixture contained 50 mM potassium phosphate buffer (pH 7.0) supplemented with 0.1 mM EDTA and 0.2 mM DHA. The reaction was triggered by the addition of 2.5 mM GSH according to the method of Nakano and Asada (1981).

### 2.11 GR (EC 1.6.4.2) activity assay

The GR activity was calculated by measuring the decrease in absorbance at 340 nm due to oxidation of NADPH (extinction coefficient: 6.22 mM^-1^ cm^-1^) as described by Donahue et al. (1997). The reaction buffer consisted of 100 mM Tris-HCl (pH 7.8), 2 mM EDTA and 0.5 mM glutathione disulfide (GSSG). The reaction was initiated by adding 0.05 mM NADPH.

### 2.12 Determination of total polyphenols, total flavonoids and total anthocyanins

For spectrophotometric analysis, 5 g of homogenized berries were extracted with 25 or 50 mL of acidified methanol (1% HCl) by mixing for one hour, then centrifuged at 10,000 *g* for 10 min at 15°C. Total polyphenols content was determined according to the Folin-Ciocalteau method (Waterhouse, 2002). One milliliter of Folin-Ciocalteau reagent, 5 mL of distilled water and 2 mL of 20% Na_2_CO_3_ were added to 0.1 mL of extract in a 20 mL volumetric flask and immediately diluted to the final volume with distilled water. The optical density, after 90 minutes, was measured at 700 nm on a UV–vis spectrophotometer (Jasco model 7800, Tokyo, Japan). Results were expressed as milligrams of gallic acid per 100 g FW. Total flavonoids were evaluated spectrophotometrically at 280 nm. A catechin standard curve was set and results were reported as milligrams of catechin per 100 grams of fresh weight (100 g FW) (Iriti et al., 2005). The total anthocyanins (ACNs) were estimated by the pH differential method (Cheng and Breen, 1991). Absorbance was measured on a UV-vis spectrophotometer (Jasco model 7800, Tokyo, Japan) at 520 nm and at 700 nm in buffers at pH 1.0 and 4.5, using the following equations:

A= [(A520 - A700) pH1.0 – (A520 – A700) pH4.5]

ACNs (mg/L) = (A x MW x DF x 1000)/(ϵ x 1)

with a molar extinction coefficient (ϵ) of malvidin-3-glucoside of 28000. Results were expressed as milligrams of malvidin-3-glucoside equivalent per 100 grams of FW.

### 2.13 Anthocyanin identification and quantification

Approximately 10 g of frozen berries were mixed with 30 mL of a solution methanol/trifluoroacetic acid (TFA) 2% in water (10:90, v/v) and homogenized by a Waring blender (Torrington, CT) with a stainless steel mini-container (37-110 mL) for 1 min. The homogenate was extracted for 30 min under agitation in the dark at room temperature. The suspension was centrifuged at 4,000 × g for 10 min at 4°C, and the supernatant recovered. The residue was extracted again until disappearance of the red color (4 × 20 mL) with a solution of methanol/TFA 2% in water (10:90, v/v), and treated as described above. The supernatants were combined, and the volume was adjusted to 200 mL by solution of 2% TFA in water. All extracts were stored at −20°C and centrifuged at 10,000 × g for 1 min before the LC analysis.

Anthocyanin identification was performed using an ACQUITY UHPLC system (Waters, Milford, MA) equipped with a model E-Lambda photodiode array detector (Waters) and an High-Resolution Mass Spectrometer Orbitrap mod. Exactive (Thermo Scientific, Rodano, Italy) equipped with an HESI-II probe for ESI and a collision cell (HCD). The separation was carried out with a C_18_ Kinetex column (150 × 4.6 mm, 2.6 μm, Phenomenex, Torrence, CA) protected with guard column, at 1.7 mL/min, and flow-rate split 5:1 before electrospray ionization (ESI) source. The column and sample were maintained at 45°C and 20°C, respectively. The eluents were (A) 0.2% TFA in water and (B) acetonitrile: 0.2% TFA in water (35:65, v/v). The linear gradient was: 0–15 min 14% B; 15–25 min from 14% to 20% B; 25–35 min from 20 to 32% B; 35–45 min from 32% to 50% B; 45–48 min 50% to 90% B; and 90% for 3 min. The MS operative conditions were: spray voltage +4.0 kV, sheath gas flow rate 60 (arbitrary units), auxiliary gas flow rate 20 (arbitrary units), capillary temperature 350°C, capillary voltage +30 V, tube lens +80 V, skimmer +25 V, and heater temperature 130°C.

The acquisition was assessed in the full-scan mode in the range (m/z)^+^ 200–2,000 u, using an isolation window of ±2 ppm. The AGC target, injection time, mass resolution, energy, and gas in the collision cell were 1 × 10^6^, 100 ms, 50 K, 20 V, and N_2_, respectively. The MS data were processed using Xcalibur Software (Thermo Scientific). Peaks were identified by evaluating the accurate mass, the fragments obtained in the collision cell, and the on-line UV spectra (220–700 nm). Working solutions (n = 5) were prepared in the range of 2–50 µg/mL, and 20 µL was injected into the chromatographic system. Anthocyanins were integrated and quantified at 520 nm. The amount of the ACNs not commercially available (D-gal, D-ara, Mv-ara and Pt-ara) was estimated using the calibration curve equation of the same anthocyanidin linked to glucose. More details have been included into **Supplementary File S1**.

### 2.14 Statistical analysis

All data were analysed for normality using the Shapiro-Wilk test (alpha = 0.05) and subjected to a two-way ANOVA. For each cultivar, significant differences among different ripening classes and treatment were calculated by Tukey’s mean test (p ≤ 0.05). Analyses were performed using GraphPad Prism version 8 for Windows (GraphPad Software, La Jolla California USA, www.graphpad.com). When no interaction was found between ripening classes and treatment (i.e. total polyphenols content), the effect of two factors was analysed independently. The detailed results of the two-way ANOVA are reported as **Supplementary File S2**.

## 3 Results

### 3.1 Fruit quality parameters

#### 3.1.1 Total titratable acidity

**Figure 3:**
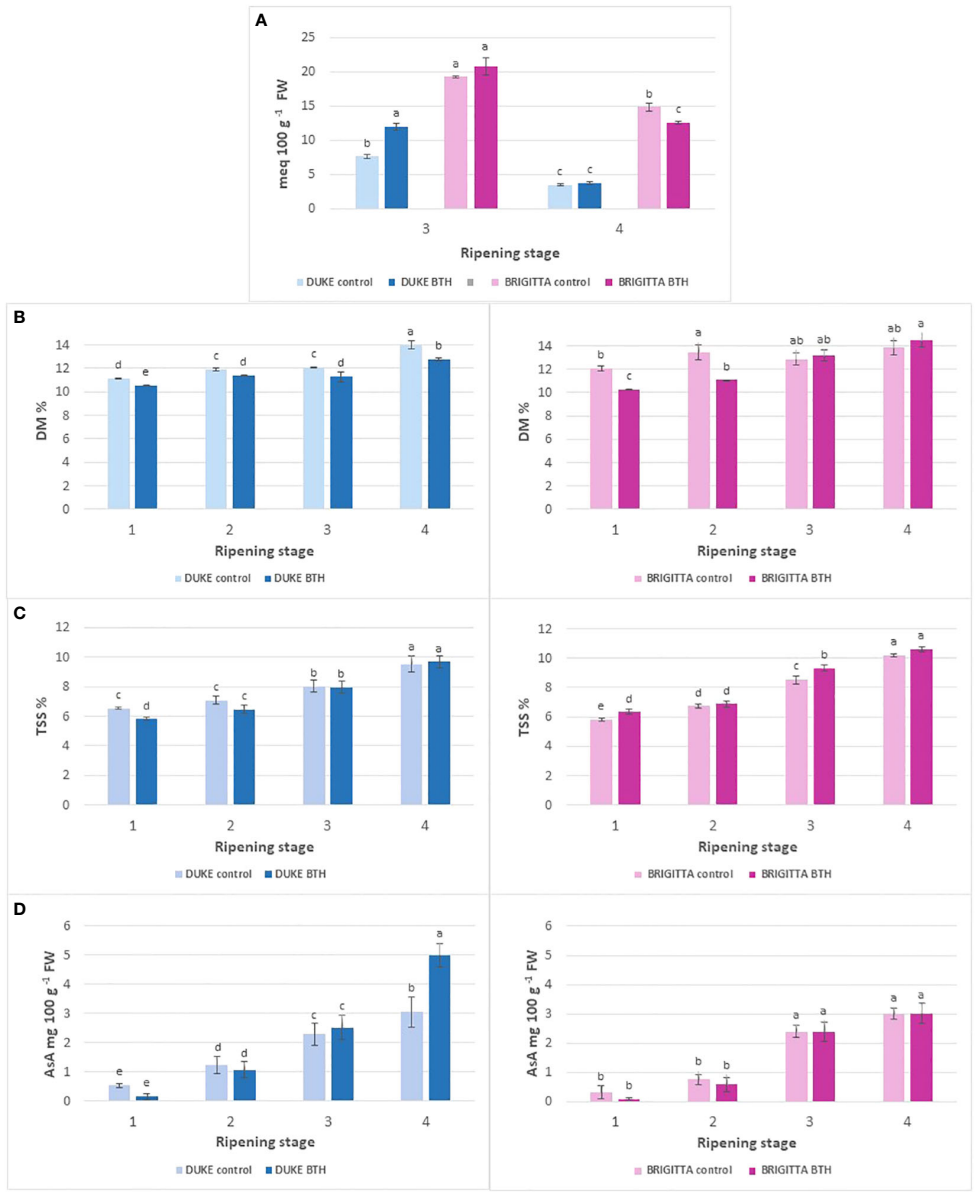
Effect of BTH treatment on **(A)** total titratable acidity and **(B)** dry matter, **(C)** total soluble solids and **(D)** ascorbic acid contents in cv ‘Duke’ (left) and ‘Brigitta’ (right). Values represent means ± SE (n = 6). Different letters indicate statistical differences (p ≤ 0.05) among ripening classes and between treatments, for each cultivar.

Titratable acidity declined most markedly in ‘Duke’ moving from third to fourth ripening stage, by 69% in treated berries and by 55% in control berries (**Figure 3A**). ‘Brigitta’ showed the highest values and maintained them even at the most advanced stage of ripening, decreasing by 39% and by 23% in treated and control fruits, respectively. BTH treatment positively affected TTA at the third stage of ripening in Duke (+57%), but not in Brigitta. On the other hand, the treatment had no effect in ripe berries of Duke, but decreased significantly the acidity in those of Brigitta (-15%).

#### 3.1.2 Analysis of dry matter

Generally, the dry matter percentage (**Figure 3B**) increased along ripening. In ‘Duke’, the dry matter percentage of berries treated with BTH showed lower values compared with controls in all ripening stages and at full ripeness it decreased by 8%. In control samples of ‘Brigitta’ the levels of dry matter measured along ripening were not statistically different, except for unpigmented berries, which had 10% lesser dry matter than berries of stage 2. thOn the other hand, in treated samples, at the end of the ripening process an increment in dry matter was detected. In this cultivar, the treatment determined lower percentage of dry weight in stages 1 and 2 but not in samples from the stages 3 and 4.

#### 3.1.3 Total soluble solids

In both the cultivars an increase of the soluble solids content throughout berry ripening was observed (**Figure 3C**). There were no significant differences between cultivars for untreated samples, on the contrary berries of ‘Duke’ treated at stage 3 and 4 with BTH had lower soluble solids content respect to the same berries of ‘Brigitta’. At the fourth ripening stage, ‘Brigitta’ and ‘Duke’ showed a similar average content of soluble solids of about 10° Brix. Treatment with BTH had significant effects on both cultivars, in different stages of maturity. It should be specified that the two cultivars, however, showed an opposite behavior: in ‘Brigitta’, the berries treated with BTH showed a higher sugar content in both the first and third stage, while in ‘Duke’ non-treated berries resulted to be richer in soluble solids at stage 1.

#### 3.1.4 AsA content

In both of the cultivars there was a sharp increment in AsA levels along ripening with the lowest levels in unripe green and purple samples (stages 1 and 2). The ascorbic acid content in ‘Duke’ and ‘Brigitta’ (**Figure 3D**) was about 2.5 mg/100 g FW in berries from stage 3, while fully mature ‘Duke’ showed higher levels compared with ‘Brigitta’ (4.2 mg/100 g FW and 3 mg/100 g FW, respectively). For each cultivar, samples from the last ripening stage showed a significant difference compared to other stages, considering both treated and non-treated berries, the only case in which there was no difference between the fourth and third stage was found in ‘Brigitta’ after treatment.

Treatment had no significant effect in ‘Brigitta’ along all the ripening process. For ‘Duke’, treatment decreased significantly AsA content in the first ripening stage, on the contrary in the fourth stage BTH exerted a positive effect and the level of AsA was significantly higher in treated berries.

### 3.2 Enzyme activities

**Figure 4:**
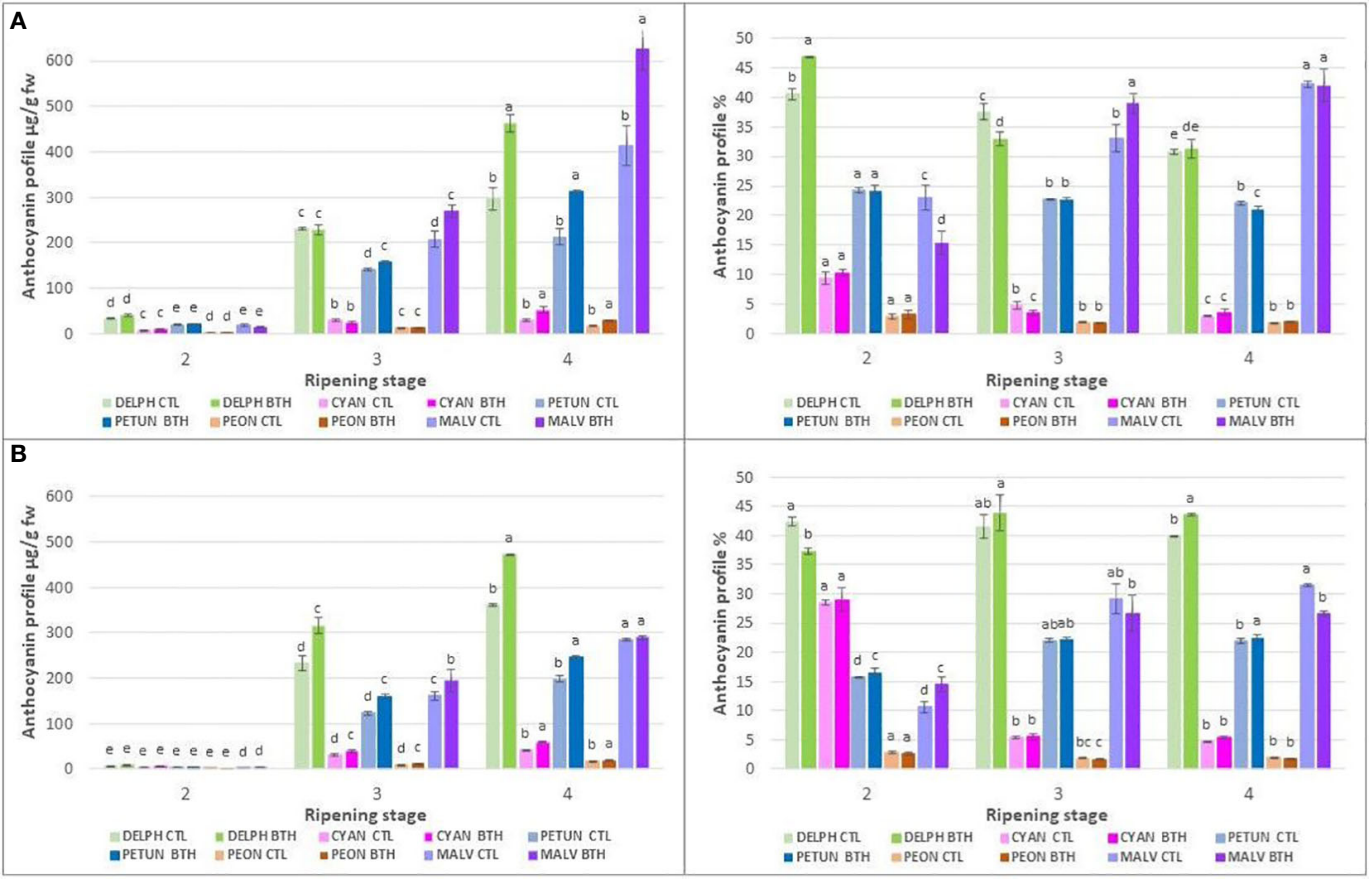
Effect of BTH treatment on total amounts (left) and relative proportion (right) of individual anthocyanins (clustered into the representative anthocyanidin classes) in cv **(A)** ‘Duke’ and **(B)** ‘Brigitta’. Values represent means ± SE (n = 6). Different letters indicate statistical differences (p ≤ 0.05) among ripening classes and between treatments, for each cultivar.

Significant differences in the activity of enzymes involved in the AsA recycling through the ascorbate-glutathione cycle were detected between blueberry cultivars and among ripening stages (**Figure 4**).

#### 3.2.1 APX

**Figure 5:**
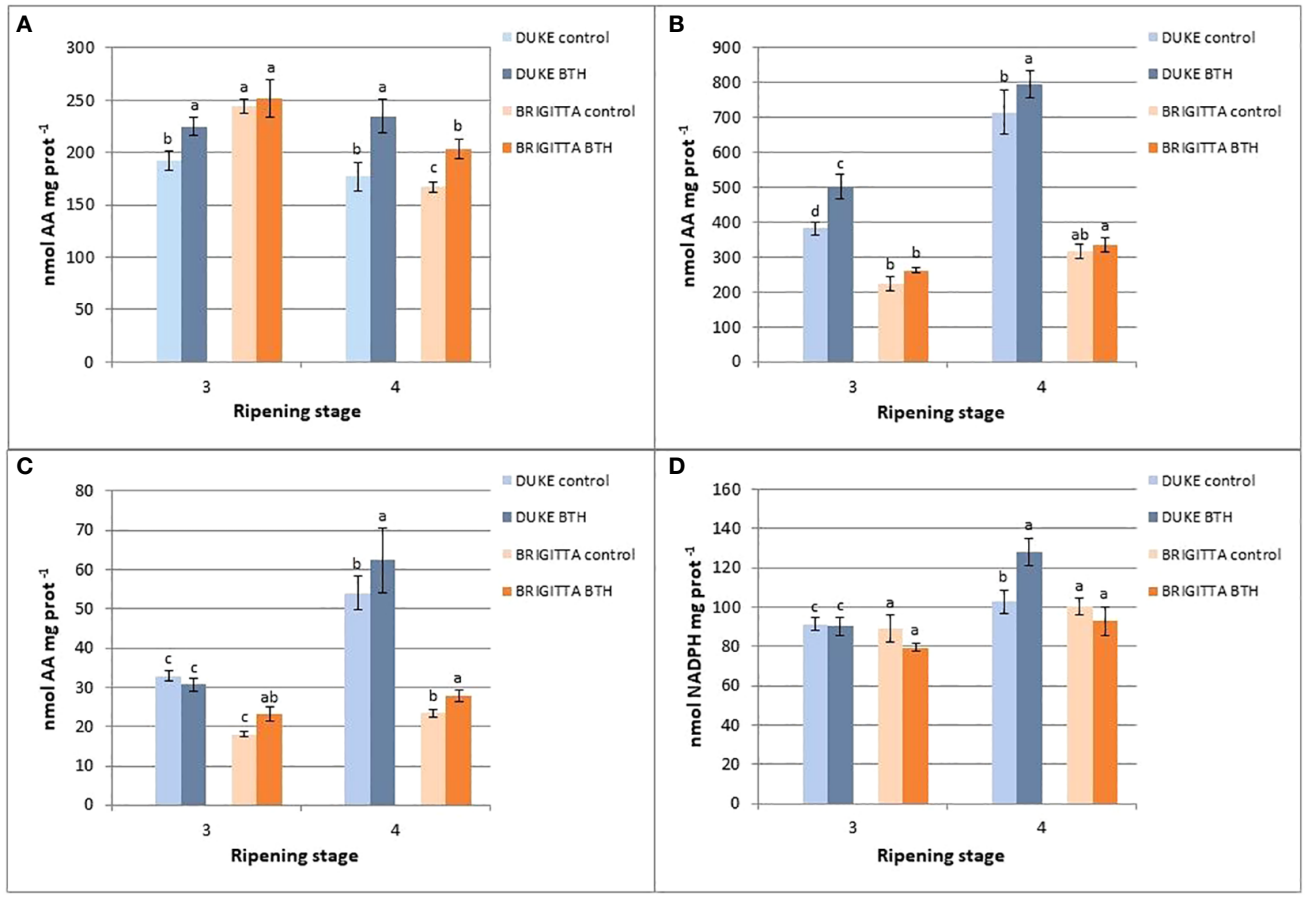
Effect of BTH on activities of **(A)** APX, **(B)** MDHAR, **(C)** DHAR and **(D)** GR in cv ‘Duke’ and ‘Brigitta’ at two ripening stages. Values represent means ± SE (n = 6). Different letters indicate statistical differences (p ≤ 0.05) among ripening classes and between treatments, for each cultivar.

APX is the first enzyme in the ascorbic acid recycling pathway. In the ripening stage 3, the specific activities were higher with respect to the last stage, in both cultivars, and ‘Brigitta’ showed slightly higher activities compared with ‘Duke’ (**Figure 5A**). At this stage, BTH had no effect on the APX activity. Along the ripening process the APX activity decreased in both, ‘Brigitta’ and ‘Duke’, but in fully ripe samples treated with BTH the values were significantly higher, i.e. by 33% in ‘Duke’ and by 22% in ‘Brigitta’.

#### 3.2.2 MDHAR

MDHAR is the enzyme that allows the regeneration of AsA from MDHA. In both the cultivars levels of activity increased from stage 3 to 4, but this pattern appeared much more evident in ‘Duke’ compared with ‘Brigitta’ (**Figure 5B**). Treatment with BTH was effective in stimulating the MDHAR activity at both stages of ripening of ‘Duke’ (stage 3: + 29%; stage 4: + 11%), while no significant differences were observed in ‘Brigitta’. Nevertheless, if considering only the effect of BTH on ‘Brigitta’ berries at stage 3, the treatment was effective in enhancing enzyme activity by 17%.

#### 3.2.3 DHAR

DHAR is the enzyme committed to catalyze the reduction of DHA to AsA by using the reducing power deriving from the oxidation of GSH. The DHAR activity was different among cultivars (**Figure 5C**). In fact, ‘Duke’ showed the highest levels of activity in both the ripening stages, but appeared to be sensitive to BTH only at full ripening with an increase of DHAR activity of 15%. On the other hand, in ‘Brigitta’ enzyme activity was lower, but was positively affected by BTH treatment showing at both stages 3 and 4 significant increments in activity values (+29% and +19%, respectively).

#### 3.2.4 GR

GR catalyzes the regeneration of oxidized GSH derived from the activity of DHAR, thus it is important in maintaining the efficiency of the recycling pathway of ascorbate. In ‘Duke’ there was an increasing trend in enzyme activity from stage 3 to 4, but not in ‘Brigitta’ (**Figure 5D**). BTH didn’t exert a marked influence on GR. Only in ‘Duke’, at the final ripening stage, BTH increased the activity levels compared to the control by 25%.

### 3.3 Total polyphenols, total flavonoids and total anthocyanins

**Figure 6:**
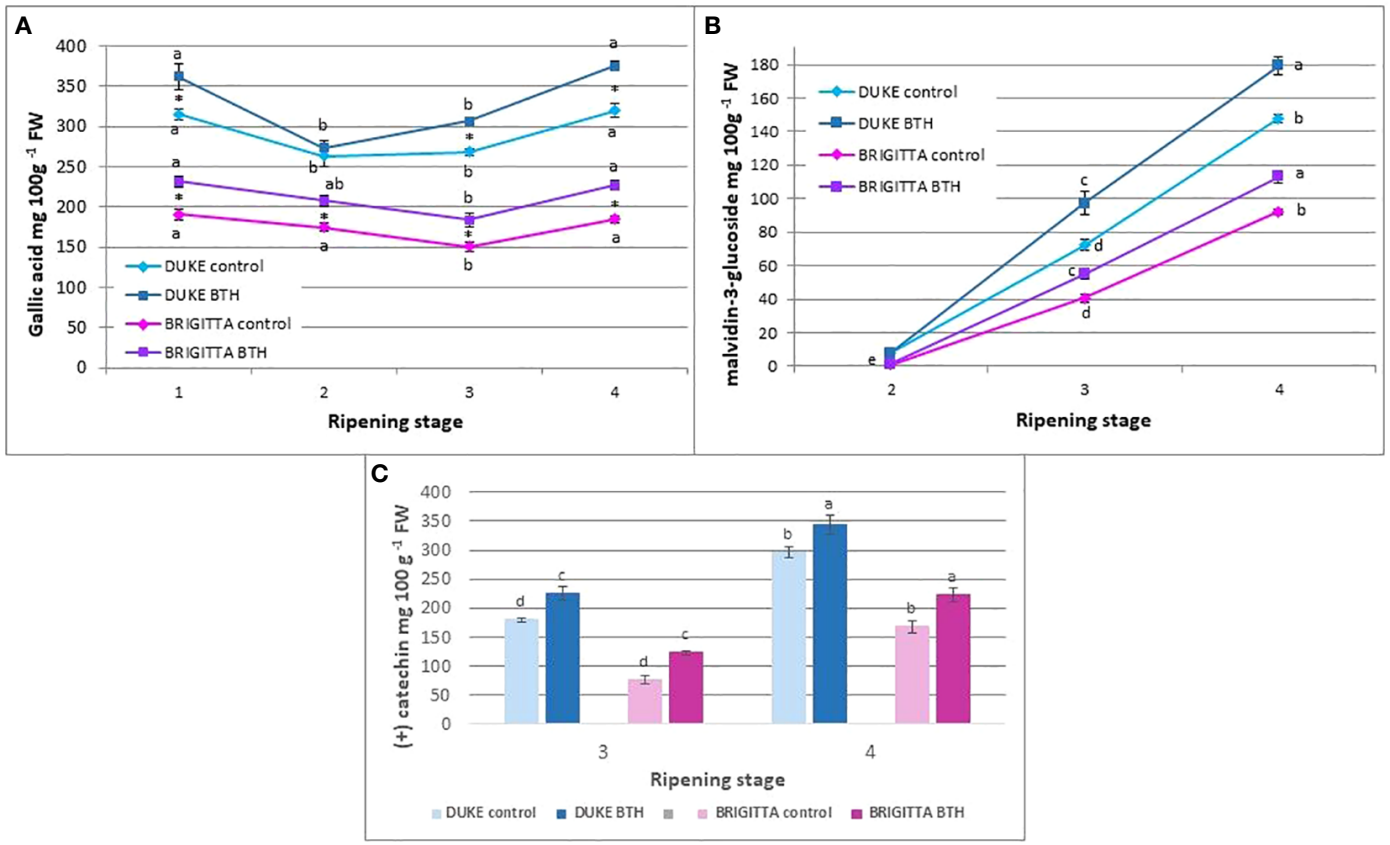
Effect of BTH treatment on **(A)** total phenolics, **(B)** total anthocyanins and **(C)** total flavonoids contents in cv ‘Duke’ and ‘Brigitta’. Values represent means ± SE (n = 6). Different letters indicate statistical differences (p ≤ 0.05) among ripening classes and between treatments for each cultivar. In **(A)**, different letters indicate statistical differences (p ≤ 0.05) among ripening classes for each cultivar, while asterisks indicate statistical differences between treatments (p ≤ 0.05).

Regarding to phenolic compounds (**Figure 6A**), the lower levels of total polyphenols were found in ‘Brigitta’, particularly at ripening stage 3 (150.5 mg/100 g FW). Along the process of ripening, ‘Duke’ maintained higher levels of polyphenols but, on the other hand, the levels decreased sharply at the stages 2 and 3 (about 270 mg/100 g FW in control plants at stage 3). In ‘Duke’ and ‘Brigitta’, during ripening, after a consistent decrease, total polyphenols increased, to reach in stage 4 the same levels as in stage 1. The BTH treatment was effective in promoting polyphenol accumulation throughout berry ripening. At all ripening stages, except for berries of ‘Duke’ at stage 2, significantly higher amounts of polyphenols were detected in treated fruits. At full ripening, BTH enhances the levels by 18% in ‘Duke’ and by 23% in ‘Brigitta’.

The content of total anthocyanin (**Figure 6B**) increased throughout berry ripening. At all the considered stages of maturity, cultivars differ significantly in anthocyanin content, with greater differences at the last ripening stage in which ‘Duke’ (164 mg/100 g FW) was confirmed to be a better source of this class of compounds compared to ‘Brigitta’ (95 mg/100 g FW).

BTH had a positive effect on anthocyanin levels in both ‘Brigitta’ and ‘Duke’, increasing the levels in berries at stage 3 by 36% and 34% and at full ripening by 23% and 21%, respectively.

The content of flavonoids (**Figure 6C**) increased during the entire period starting from the early phases to the end of the ripening process and at the last stage the higher levels were registered in both of the cultivars. ‘Duke’ showed higher concentrations compared with ‘Brigitta’, confirming this cultivar as the better source of polyphenols and the richest in flavonoids (319.4 mg/100 g FW at stage 4).

Treatment with BTH promoted in both cv the accumulation of total flavonoids during the last stages of berry ripening, enhancing the levels at stages 3 and 4 of ‘Brigitta’ by 61% and 33%, respectively, and by 25% and 16% at the same stages of ‘Duke’.

### 3.4 Anthocyanin profile

Anthocyanin profiles of ‘Duke’ and ‘Brigitta’ were systematically analyzed over the ripening process starting from the beginning of fruit pigment accumulation at stage 2. The anthocyanins identified were monoarabinosides (ara), monogalactosides (gal) and monoglucosides (glu) of delphinidins (Dp), cyanidins (Cy), petunidins (Pt), peonidins (Pn), and malvidins (Mv). During ripening, the main anthocyanidins that accumulated in ‘Duke’ berries were Mv and Dp followed by Pt. In ‘Brigitta’ berries, the predominant anthocyanidin was Dp, subsequently Mv and Pt. As in ‘Duke’, Cy and Pn were in minor proportion.

In both cultivars, the tri-hydroxylated (in the lateral B ring) Dp, Mv, Pt and the di-hydroxylated Cy and Pn were equally conjugated with gal and ara. During berry development, anthocyanin levels progressively and steadily increased (**Figure 4**). Consequently, the individual anthocyanin amounts were significantly different among ripening classes.

The total amount of anthocyanins, calculated as the sum of all the individual anthocyanins resulting by the chromatographic profiles, showed significant differences among all the classes of ripening, confirming the results obtained by the spectrophotometrical analysis.

BTH treatment had strong influence on anthocyanin accumulation (**Figure 4** left) In terms of absolute concentrations, BTH enhances the levels of all the anthocyanins in ripe berries of both cultivars, except Mv in ‘Brigitta’ (**Figure 4B**, left). After treatment, the highest anthocyanins increment in ripe berries regarded Dp (+ 56%), Pt (+ 46%) and Mv (+ 52%) for ‘Duke’ and Dp (+ 31%) and Pt (+ 56%) for ‘Brigitta’. Moreover, a marked positive response of tri-substituted pigments, i.e. Pt (+ 11%) and Mv (+ 29%) in ‘Duke’ (**Figure 4A**, left) and Pt (+ 30%) and Dp (+ 36%) in ‘Brigitta’ (**Figure 4B**, left), was evident also in berries of stage 3. In addition to absolute concentrations, it is useful to express anthocyanin composition in relative terms (**Figure 4** right). Considering the relative proportions of anthocyanins in ripe berries, in ‘Duke’ (**Figure 4A**, right) BTH decreased only the percentage of Pt (- 4%), whereas in ‘Brigitta’ (**Figure 4B**, right) the effect of treatment was more evident: considering the tri-substituted pigments the increase in Dp (+ 9%) partially occurred at the expense of Mv (- 15%) and considering the di-substituted ones, BTH determined the increase in Cy (+ 14%) concomitant with the decrease of Pn (- 8%). In berries of stage 3, the treatment enhanced the percentage of Mv (+ 18%) and reduced that of Dp (- 12%) in ‘Duke’, while it had no effect in ‘Brigitta’.

**Figure 7:**
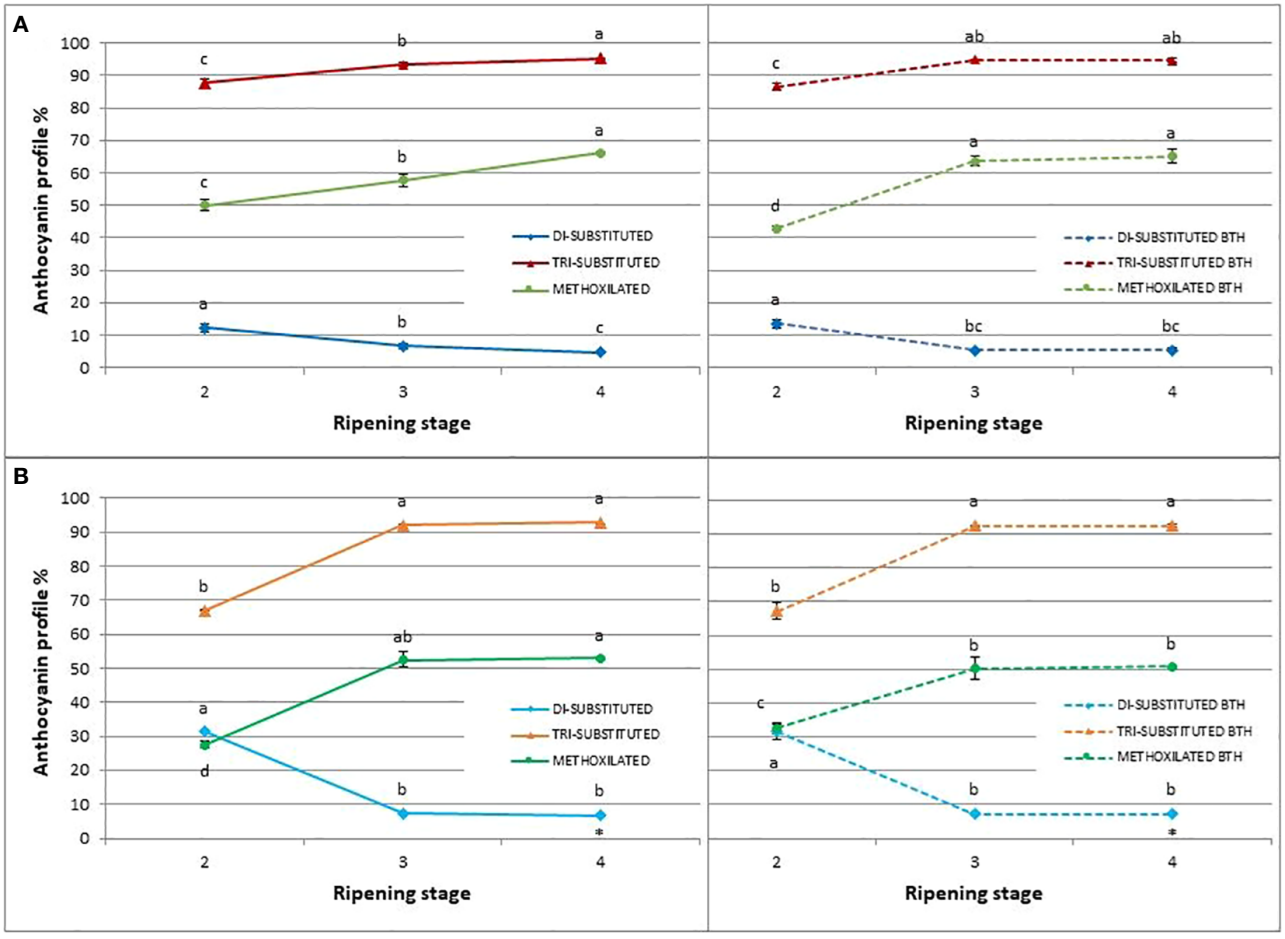
Effect of BTH treatment on relative proportion of di-substituted anthocyanins, tri-substituted anthocyanins, and methoxilated anthocyanins (%) in control (left) and treated (right) cv. **(A)** ‘Duke’ and **(B)** ‘Brigitta’. Values represent means ± SE (n = 6). Different letters indicate statistical differences (p ≤ 0.05) among ripening classes and between control (left) and treatment (right), for each cultivar.

Regarding the relative proportion of the individual anthocyanins during blueberry ripening (**Figure 7**), a shift of anthocyanin biosynthesis from Cy-type, di-substituted molecular structures toward Dp-based, tri-substituted pigments was evident. Marked decreases in the proportions of di-substituted anthocyanins and increases in the proportions of tri-substituted forms were observed between stages 2 and 3 of berry ripening particularly in ‘Brigitta’ (- 76% and + 37%, respectively) and to a lesser extent in ‘Duke’ (- 45% and + 6%, respectively). Then, the relative ratios were maintained during the later phase of ripening in ‘Brigitta’ whereas changed following the trend of the previous ripening stages in ‘Duke’. In both cv ‘Brigitta’ BTH did not alter this pattern.

Regarding the methoxilated derivatives of anthocyanins, i.e Pn derived from Cy and both Pt and Mv derived from Dp by the action of O-methyltransferase, BTH increased its levels at stage 2 (+18%) and decreased them at stage 4 (- 7%) in ‘Brigitta’. In ‘Duke’, after a decrease at the stage 2 (-14%), higher concentrations were promoted in treated berries at stage 3 (+10%).

## 4 Discussion

BTH exogenous application can mimic the action of the signaling molecule SA and simulate the attack of a pathogen. It may interact with receptors in the plant, activating defense responses. For this reason, elicitors were primary used to improve plant resistance against pathogens. These compounds do not kill pathogens but trigger plant defense mechanisms, among them, the production of increased levels of phenolic compounds.

In our study we analysed how BTH preharvest treatments may affect blueberry fruit quality during fruit ripening as well as at harvest, in two different cv, ‘Brigitta’ and ‘Duke’. We considered some bioactive/health related antioxidants, i.e AsA and polyphenols, which are important both for human consumption (at fruit ripeness) and for plant physiological responses to biotic and abiotic stresses (during fruit development).

AsA contributes to cellular redox balance during stress conditions that increase ROS production, including SAR induction.

In fact, AsA reflects the short-term reaction of the plant immune response and phenolic compounds represent products of the plant secondary metabolism, which can be induced by SAR, after BTH treatment. AsA could have low nutritional importance in blueberries due to its low concentration, but is of physiological relevance in the SAR response and redox homeostasis.

The increasing trend in dry matter percentage along blueberry ripening is consistent with data previously reported for blueberry (Mannozzi et al., 2017) and is slightly affected by BTH treatment at the last stage of ripening only in ‘Duke’.

TTA and soluble solids content are both important indexes of ripening. ‘Brigitta’ had higher levels of TTA compared to ‘Duke’ and maintained them even at the most advanced stage of ripening (stage 4). This feature could be linked to the good attitude of ‘Brigitta’ to storage. TTA decreases during ripening, as also reported in other studies on *V. corymbosum*, L. (Castrejón et al., 2008). In both the cultivars the increase of the soluble solids content throughout berry ripening is consistent with data previously published (Castrejón et al., 2008). BTH exerted different effects on TTA and TSS, depending on ripening stage and cv. An enhancement of total acidity occurred in ‘Duke’ at the stage 3 and a decrease at full ripening in ‘Brigitta’. Likewise, TSS content did not change at the later stages (3 and 4) of ripening in ‘Duke’, whereas treated berries of ‘Brigitta’ showed at stage 3 higher amounts of TSS compared to control. Thus, the ripening pattern of ‘Duke’ and ‘Brigitta’ is different in response to BTH, as confirmed also by the data on dry matter content. These results are in accordance with those reported by Cocetta et al. (2015), who showed a genotypic-dependent variation in TSS amount between blueberry cultivars ‘Duke’ and ‘Blueray’ in response to methyl jasmonate treatment. On the other hand, Wang et al. (2018) reported that methyl jasmonate did not alter the ripening pattern of two rabbiteye blueberry cultivars.

Moreover, our data partially differ from those reported by other authors. Cao and Jiang (2006) showed that the application of BTH on trees during fruit growth did not affect the edible quality of Yali pears; Liu et al. (2005) found no effects of peach postharvest treatment with BTH on fruit quality (TTA and TSS), while Li et al. (2015) highlited a significant decline in TA contents induced by BTH in muskmelon during storage, but no substantial changes regarding TSS contents.

Antioxidant compounds play a pivotal role in defense against ROS,protecting plants against oxidative stress damage. In plants, an higher content of antioxidants substantially increase the resistance to abiotic and biotic stresses. Plants have sophisticated antioxidant defense systems to copy with ROS damage, including both efficient enzymatic (such as APX, MDHAR, DHAR, GR, SOD, etc.) and nonenzymatic (e.g. AsA, phenolic compounds, GSH). AsA, as an antioxidant, is capable to accept electrons from a wide range of free radicals and in this activity, it undergoes enzymatic regeneration from its oxidized forms, MDHA and DHA. It is recognized that increased AsA levels through enhanced recycling could ensure a greater direct protection against free radicals than through increased biosynthesis (Mellidou and Kanellis, 2017).

AsA content measured for ‘Duke’ and ‘Brigitta’ differed at full ripening. Variability in the concentration of ascorbic acid between cultivars was also reported in a study on two blueberry cultivars, ‘Darrow’ and ‘Bluecrop’, which investigated the effect of the genetic diversity and of date of harvest on ascorbate and phenolic compounds (Lata et al., 2005), and also by Prior et al. (1998) in their studies on *Vaccinium* spp. Moreover, data on AsA content in ‘Duke’ and in ‘Brigitta’ were slightly different from what reported by other authors. For example, higher contents of ascorbic acid were reported by Prior et al. (1998) and by Shivembe and Ojinnaka (2017) for ripe berries from the cultivar ‘Duke’ (7.3 mg/100 g FW and 9.8 mg/100 g FW, respectively) and by Golding et al. (2014) for ‘Brigitta’ (10.17 mg/100 g FW). This may depend on many factors which can affect the accumulation of this compound, such as different climatic conditions, cultural practices and different stage of ripening at harvest. Moreover, the sharp increment in AsA levels along ripening of ‘Duke’ and ‘Brigitta’ berries is quite different compared with what is observed during development and ripening of bilberry (*Vaccinium myrtillus* L), in which the levels of AsA do not change dramatically and show the highest levels at the first ripening stage (Cocetta et al., 2012). BTH did not influence the levels of AsA in ‘Brigitta’, consistently to the data reported by Liu et al. (2005) for peach, but increased them in ‘Duke’ as showed by Wei et al. (2019) in apples and by Ge et al. (2019) in blueberry cv ‘Blue Gold’, during storage. AsA is often used as a reliable biochemical marker of freshness for horticultural produces. Studies performed on apples (Wei et al., 2019), and strawberries (Romanazzi et al., 2013) have shown that senescence of plant tissues was associated with AsA decline; the observed reduction of oxidative stress in some fruits could be one of the reasons why BTH-based treatment is useful in delaying senescence phenomena (Li et al., 2015; Li et al., 2020a; Xu et al., 2021).

In our work, BTH was effective in stimulating the activities of enzymes involved in the AsA recycling, but the two cultivars reacted differently to treatment at different ripening stages.

APX is the first enzyme in the cycle, it shows high affinity for AsA as a substrate and is very important in the detoxification of H_2_O_2_. BTH is thought to inhibit the activity of APX (Wendehenne et al., 1998), leading to a burst in H_2_O_2_ production. Our data are not supporting the hypothesis of an inhibitory effect on the activity of APX by BTH. On the contrary, BTH exerted a positive effect on both cultivars at full ripening stage. We have not measured the levels of H_2_O_2_ but, since we collected samples 24 hours after treatment and treatments were repeated starting from the beginning of fruit pigmentation throughout the harvest season, it is possible to speculate that after an initial suppression of the APX activity, the high levels of H_2_O_2_ lead to an increment in the enzymatic activity as we observed in full ripe samples. De Pinto et al. (2006) pointed out the relevance of different timing and H_2_O_2_ levels, as critical points for APX behaviour. The constant production of low amounts of ROS determines a transient rise in APX. Such a rise is aimed at restoring redox impairment due to H_2_O_2_ overproduction. An activation of the antioxidant metabolism has been reported widely in the literature as a first line of defence against moderate oxidative stress (Shigeoka et al., 2002; Vranová et al., 2002). It is likely that the regulation of APX expression and activity is part of a mechanism for controlling the balance between beneficial and detrimental roles of H_2_O_2_ in plant cells. A positive effect of BTH on APX activity is also reported by other authors in blueberries (Ge et al., 2019), apples (Wei et al., 2019), pears (Li et al., 2020b), and muskmelons (Ge et al., 2015). On the contrary, APX activity in young Yali pears was temporally decreased by BTH sprays on trees during fruit growth, but this temporary decrease is assumed by the authors to contribute to the increase of H_2_O_2_ content in plant tissues, an event considered to be a component of the mechanism of the BTH-enhanced disease resistance in pear fruit (Cao and Jiang, 2006).

MDHAR catalyzes the reduction from MDHA to AsA. Rapid regeneration of AsA is necessary to maintain its antioxidant potential (Wang et al., 2011). MDHAR was reported to be influenced by many stresses and treatments. For example, exposure of mature green tomatoes to low oxygen, regardless of concentration, resulted in a decline of MDHAR transcript levels after 1 hour (Ioannidi et al., 2009). Stevens et al. (2008) showed that MDHAR activity levels correlate with reduced AsA levels in tomato fruit in presence of chilling stress condition. From our results, MDHAR activity appeared to be positively correlated with AsA accumulation in the last two ripening stages. This is in line with the results reported by Liu et al. (2015) in blueberry, Cruz-Rus et al. (2011) in strawberry and Liang et al. (2017) in sweet cherry. BTH was effective in promoting MDHAR activity in both cv at the third stage and only in ‘Duke’ at full ripening. An enhanced MDHAR activity after BTH treatment is also described in ‘Docteur Jules Guyot’ pears (Li et al., 2020a) and in the soluble fraction of pea leaves (Clemente-Moreno et al., 2010). Moreover, the study of Wei et al. (2019) on apple fruit indicated that BTH treatment stimulated MDHAR enzymatic activity but meanwhile it inhibited the MDHAR gene expression. As the MDHAR gene was demonstrated to be regulated at transcriptional level in acerola, at a temperature of 4°C, under NaCl stress condition (Eltelib et al., 2011), the authors speculated that MDHAR gene in the BTH-treated apple is regulated at posttranscriptional level.

DHAR catalyzes the reduction of DHA to AsA *via* the oxidation of GSH. Thus, DHAR represents a valuable component implicated in the protection of cells from oxidative stress. Increased DHAR activity was reported in response to several ROS-inducing stresses, such as hydrogen peroxide, ozone, salt, drought, and low temperature (Eltayeb et al., 2006). Our study revealed that DHAR displayed an opposite trend compared to MDHAR, namely BTH stimulated DHAR activity in fully ripe blueberries of both cv, but only in ‘Brigitta’ at the third stage of ripening. The increase in DHAR activity in response to BTH application is a common feature in plant tissues, such as apples (Wei et al., 2019) and pears (Li et al., 2020a). MDHAR and DHAR are mainly deputed to the maintenance of the dynamic balance in AsA content, and interestingly in our study the activation of the two enzymes by BTH occurred in both cv at two different stages of blueberry ripening. Thus, these data imply the involvement of the two enzymes in the enhanced regeneration of AsA at two subsequent phases of the ripening process, but only in ‘Duke’ it led to an increase in ASA content. These responses are likely due to the different genetic background of blueberry cultivars (Mellidou and Kanellis, 2017). Similar results on genetic variability in regard to AsA accumulation were reported by Liu et al. (2015) for ‘Bluecrop’ and ‘Berkley’ blueberries, and by Chiaiese et al. (2019) in two varieties of pepper with low and high antioxidants content. Moreover, Totad et al. (2020) reported intraspecific variation considering a set of nutritional and functional attributes of eight blueberry varieties.

GR plays a role in AsA recycling pathway, as it reduces the oxidized GSH derived from the activity of DHAR. BTH has been reported to increase the activity of GR in strawberry fruits (Cao and Jiang, 2006) and muskmelon (Ge et al., 2015) during storage, in pea leaves (Clemente-Moreno et al., 2010), in apples (Wei et al., 2019) and pears (Li et al., 2020a). In our experiment we did not observe great variations in GR activity and the values we recorded were quite similar between cultivars and at different ripening stages. The only significant change was observed in ‘Duke’ at the last stage of ripening when the levels increased in treated berries compared with the control. Hence, the response of the recycling pathway of AsA to BTH was slightly different in fully ripe berries of ‘Duke’ compared to ‘Brigitta’, and it could have contributed to the higher AsA content in ‘Duke’. In fact, a previous study revealed a positive correlation between GR activity and AsA levels (Wang et al., 2011).

Moreover, activities of all enzymes of the recycling pathway of AsA were positively affected by BTH treatment in ‘Duke’ at full ripening, whereas in ‘Brigitta’ at the same stage only those of APX and DHAR were stimulated. The different AsA contents in the two cv could also be related to differences in these enzyme activities.

The stress response triggered by BTH can result in an activation of the secondary metabolic pathway that leads to accumulation of phenolic compounds. Treatments with BTH were reported to be effective in enhancing PAL activity and increasing the levels of total phenols, flavonoids and anthocyanins (Cao et al., 2010).

The amount of total polyphenols that we measured is comparable with those reported by Gavrilova et al. (2011) for ‘Duke’ and by Spinardi et al. (2019) for ‘Brigitta’. Moreover, differences among our data and results published in other studies on blueberry cultivars prove that polyphenol content depends on genetic background, as previously suggested (Prior et al., 1998; Taruscio et al., 2004). Contribution to blueberry total polyphenols content is mainly due to the phenolic acid group of hydroxycinnamic acids derivatives and to the flavonoid subclasses of flavanols (and their polymers proanthocyanidins), flavonols, and anthocyanins. Flavonols, proanthocyanidins, and hydroxycinnamic acids are present in higher concentrations in young fruits. On the contrary, the contents of these compounds considerably decrease at later stages of berry development, when the accumulation of anthocyanins progressively increases during ripening, and they represent the major flavonoids in the ripe blueberry (Castrejón et al., 2008; Zifkin et al., 2012), as well as in ripe bilberry (Karppinen et al., 2016). Polyphenolic level therefore integrates all the different accumulation patterns of these classes of compounds. BTH effectively increased the levels of polyphenols in both cv throughout berry ripening. At all ripening stages, except for berries of ‘Duke’ at stage 2, significantly higher amounts of total polyphenols were detected in treated fruits. At full ripening, BTH enhances the levels by 18% in ‘Duke’ and by 23% in ‘Brigitta’.

A valuable part of the pool of phenolic compounds, in blueberry, is represented by flavonoids. In this study, the amounts of total flavonoids and the effect of BTH were investigated in the two blueberry cv at the last two stages of ripening (**Figure 4**). The levels we found are comparable with those reported for blueberry in previous studies (Cocetta et al., 2015; Spinardi et al., 2019), although in the present study the accumulation of flavonoids increased progressively throughout ripening in accordance to Guofang et al. (2019), whereas other authors reported a decline (Aires et al., 2017; Sun et al., 2018). As for the total polyphenols, also for total flavonoids the BTH treatment promoted an increase in the amounts in both cv at all the stages considered.

Among phenolic compounds, the flavonoid subclass of anthocyanins is one of the most interesting and representative in blueberry. The content of total anthocyanin increased starting from the breaker stage (stage 2), as reported by many authors for blueberry and several other *Vaccinium* species (Jaakola et al., 2002; Spinardi et al., 2019). The analysis of the amount of anthocyanins in unripe green berries (stage 1) was not performed, as those pigments were not detectable. In fact, unripe berries were reported to completely lack of anthocyanins (Kalt et al., 2003). The pattern of accumulation of total flavonoids and total anthocyanins were very similar, suggesting that, starting from color break, anthocyanins represent the major class of flavonoids. BTH stimulated anthocyanin accumulation significantly throughout the ripening period in both ‘Duke’ and ‘Brigitta’, in accordance with the data on total polyphenols and flavonoids. Similar results have been reported in blueberries cv. ‘Blue Gold’ after BTH postharvest treatment (Ge et al., 2019), in strawberry (Cao et al., 2010 and Cao et al., 2011), and in grape (Iriti et al., 2004). To shed more light on the metabolism of anthocyanins, the total amount and the relative abundance of individual anthocyanins were also considered. Considering anthocyanin metabolism, dihydrokaempferol represents the branching point between two different anthocyanin downstream pathways. Flavonoid 3’-hydroxylase (F3’H), converts dihydrokaempferol to dihydroquercetin and supports the biosynthesis of Cy and Pn glycosides (Cy-type anthocyanins), while flavonoid 3’,5’ hydroxylase (F3’5’H) gives rise to dihydromyricetin and downstream to Dp, Pt and Mv glycosides (Dp-type anthocyanins). BTH strongly stimulated the accumulation of the different forms of anthocyanins: in ‘Duke’, especially Mv and Pt, whereas in ‘Brigitta’ mainly Dp and Pt. An increase of tri-hydroxylated anthocyanins was reported after different stresses perception, such as drought conditions (Castellarin et al., 2007; Ollé et al., 2011) or high temperatures (Zhu et al., 2017; Yan et al., 2020) during ripening in grape berries, although the metabolic response and change in anthocyanin composition could vary relating to the magnitude of stress (Rienth et al., 2021). On the other hand, anthocyanin composition was not deeply altered by BTH treatment and the increase in percentage of an anthocyanin was counterbalanced by the decrease of one of the same substitution group (e.g. in ripe berries of ‘Brigitta’, Dp increase vs Mv decrease, Cy increase vs Pn decrease; in berries of ‘Duke’ at the third stage, Mv increase vs Dp decrease). Consequently, BTH had a positive impact on the concentrations of almost all pigments, with only secondary effects the relative proportions. Moreover, considering the anthocyanin profile variations during ripening, the relative proportion of di-substituted pigments decreased, while the tri-substituted forms became predominant. As well, the anthocyanin methoxylation was stimulated. These changes during berry ripening have been reported by many authors in grape (Degu et al., 2014; De Lorenzis et al., 2016).

As in grape, also in blueberry a Dp-type anthocyanin profile enrichment towards the last stages of ripening has been observed (Spinardi et al., 2019). This pattern of changes in the proportion of anthocyanins grouped by substitutions, during berry ripening, was not altered by BTH, thus the treatment didn’t impact on the modification in pigment composition related to development.

## 5 Conclusions

The purpose of the present study was to evaluate the effect of BTH treatment on two blueberry cultivars during ripening. Analyses were directed to the study of accumulation of health-promoting compounds (including polyphenols, anthocyanins, flavonoids and ascorbic acid), essential for human diet and also crucial during the entire fruit development for the plant response to biotic and abiotic stresses. Other crucial parameters were considered, as some indices of maturity and sensorial quality (including soluble solids and titratable acidity). Moreover, the effect of BTH application on the enzymatic system committed in the detoxification from H_2_O_2_, by measuring the specific activities of APX, MDHAR, DHAR, and GR was studied.

Blueberry is confirmed to be a high-valuable nutraceutical produce, however it shows significant differences in antioxidants content depending on genetic diversity and in response to different cultivation practices.

In this study, BTH stimulated the accumulation of anthocyanins and of total polyphenols and flavonoids, in the last stages of ripening. It was also active in stimulating the enzymatic mechanisms of response to oxidative stress. The increase in content of bioactive compounds in fruits represent an interesting challenge which can lead to beneficial effects on human health, as well as on qualitative aspects of the fruits, such as color or storage performance. Furthermore, an increase in the levels of antioxidants is a prerequisite to ensure a longer storage and shelf life of the fruit. From our results, in blueberry, an activation of secondary metabolism, with a consequent increase in polyphenolic compounds, did not affect the primary metabolites accumulation such as soluble solids and organic acids, which were not negatively affected following the treatment. It is important to point out how different cultivars behave differently during ripening and, even more, in response to treatment. In fact, in many cases ‘Duke’ and ‘Brigitta’ showed different or even opposite behavior after treatment with BTH, suggesting that the responses are modulated differently according to genotypes.

The obtained results could be useful in breeding programs aiming to develop cultivars with optimal traits in terms of health-promoting components and quality attributes.

## Data availability statement

The raw data supporting the conclusions of this article will be made available by the authors, without undue reservation.

## Author contributions

AS: substantial contributions to the conception or design of the work GC, AS, RB, BC: acquisition, analysis, and interpretation of data for the work AS, GC: drafting the work BC, RB: critical revision of the manuscript. All authors contributed to the article and approved the submitted version.
