# Supplementary material for: Benzothiadiazole enhances ascorbate recycling and polyphenols accumulation in blueberry in a cultivar-dependent manner
Source: Front Plant Sci. 2022 Dec 9;13:1032133. doi: 10.3389/fpls.2022.1032133 (PMC9780449; doi:10.3389/fpls.2022.1032133)
Supplement: Supplementary file 1 [file DataSheet_1.pdf]

Supplemental

Chromatograms integrated at 520 nm of blueberry fruits at higher content in glyosylated anthocyanidins.

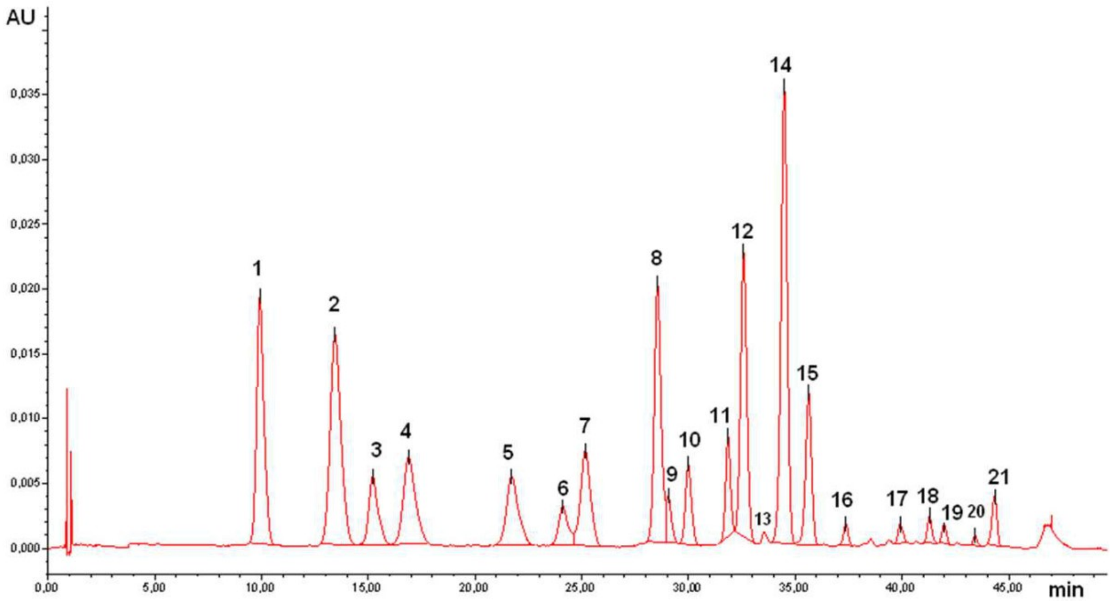

Compounds identified in the analyzed samples. For each compound is reported the retention time (RT, min), λ_{max} (nm), HR-Mass of the M^+, molecular formula and fragment ions M^+.

Peak	RT (min)	λ_{max}	$[M]^+$	Formula	Fragment ion $[M]^+$	Formula	Peak identification
1	10,0	515	465,1026	$C_{21}H_{21}O_{12}$	303,0499	$C_{15}H_{11}O_7$	D-gal
2	14,0	515	465,1026	$C_{21}H_{21}O_{12}$	303,0500	$C_{15}H_{11}O_7$	D-glc
3	15,0	515	449,1079	$C_{21}H_{21}O_{11}$	287,0548	$C_{15}H_{11}O_6$	Cy-gal
4	17,0	515	435,0920	$C_{20}H_{19}O_{11}$	303,0500	$C_{15}H_{11}O_7$	D-ara
5	22,0	515	449,1079	$C_{21}H_{21}O_{11}$	287,0548	$C_{15}H_{11}O_6$	Cy-glc
6	24,0	515	419,1000	$C_{20}H_{19}O_{10}$	287,0548	$C_{15}H_{11}O_6$	Cy-ara
7	25,0	515	479,1180	$C_{22}H_{23}O_{12}$	317,0655	$C_{16}H_{13}O_7$	Pet-gal
8	28,5	515	479,1180	$C_{21}H_{21}O_{12}$	317,0655	$C_{16}H_{13}O_7$	Pet-glc
9	29,0	515	463,1232	$C_{22}H_{23}O_{11}$	301,0705	$C_{13}H_{13}O_6$	Peo-gal
10	30,2	515	433,1127	$C_{21}H_{21}O_{11}$	317,0655	$C_{16}H_{13}O_7$	Pet-ara
11	32,0	515	463,1232	$C_{22}H_{23}O_{11}$	301,0705	$C_{13}H_{13}O_6$	Peo-glc
12	32,5	515	493,1339	$C_{23}H_{25}O_{12}$	331,0810	$C_{17}H_{15}O_7$	Mv-gal
13	33,2	515	433,1128	$C_{21}H_{21}O_{10}$	301,0705	$C_{13}H_{13}O_6$	Peo-ara
14	34,5	515	493,1339	$C_{23}H_{25}O_{12}$	331,0810	$C_{17}H_{15}O_7$	Mv-glc
15	35,4	515	463,1231	$C_{22}H_{23}O_{11}$	331,0810	$C_{17}H_{15}O_7$	Mv-ara
16	37,0	515	507,1127	$C_{23}H_{23}O_{13}$	303,0490	$C_{15}H_{11}O_7$	D-Hex-Ac
17	40,0	515	491,1180	$C_{23}H_{23}O_{12}$	287,0548	$C_{15}H_{11}O_6$	Cy-Hex-Ac
18	41,0	515	521,1280	$C_{24}H_{25}O_{13}$	317,0655	$C_{16}H_{13}O_7$	Pet-Hex-Ac
19	42,0	515	535,1444	$C_{25}H_{27}O_{13}$	331,0810	$C_{17}H_{15}O_7$	Mv-gal-Ac
20	43,4	515	505,1330	$C_{24}H_{25}O_{12}$	301,0705	$C_{13}H_{13}O_6$	Peo-Hex-Ac
21	44,5	515	535,1445	$C_{25}H_{27}O_{13}$	331,0810	$C_{17}H_{15}O_7$	Mv-glc-Ac

D: Delphinidin, Cy: Cyanidin, Pet: Petunidin, Peo: Peonidin, Mv: Malvidin, gal: galactose, glc: glucose, ara: arabinose, Hex: Hexose, Ac: acetate.
