# Supplementary material for: Benzothiadiazole enhances ascorbate recycling and polyphenols accumulation in blueberry in a cultivar-dependent manner
Source: Front Plant Sci. 2022 Dec 9;13:1032133. doi: 10.3389/fpls.2022.1032133 (PMC9780449; doi:10.3389/fpls.2022.1032133)
Supplement: Supplementary file 2 [file DataSheet_2.pdf]

Statistic output of the two-way anova

Fig. 3A

TTA DUKE			
Source of Variation	P value	P value summary	
Interaction	0,0054	**	
RIPENING STAGE	<0.0001	****	
TREATMENT	0,0142	*	

TTA BRIGITTA			
Source of Variation	P value	P value summary	
Interaction	0,0037	**	
RIPENING STAGE	<0.0001	****	
TREATMENT	0,4728	ns	

Fig. 3B

DM DUKE			
Source of Variation	P value	P value summary	
Interaction	0,0007	***	
RIPENING STAGE	<0.0001	****	
TREATMENT	<0.0001	****	

DM BRIGITTA			
Source of Variation	P value	P value summary	
Interaction	0,0037	**	
RIPENING STAGE	<0.0001	****	
TREATMENT	0,0162	*	

Fig. 3C

TSS DUKE			
Source of Variation	P value	P value summary	
Interaction	0,0089	**	
RIPENING STAGE	<0.0001	****	
TREATMENT	0,0025	**	

TSS BRIGITTA			
Source of Variation	P value	P value summary	
Interaction	0,3143	ns	
RIPENING STAGE	<0.0001	****	
TREATMENT	0,0005	***	

Fig. 3D

AsA DUKE			
Source of Variation	P value	P value summary	
Interaction	<0.0001	****	
RIPENING STAGE	<0.0001	****	
TREATMENT	<0.0001	****	

AsA BRIGITTA			
Source of Variation	P value	P value summary	
Interaction	0,9509	ns	
RIPENING STAGE	<0.0001	****	
TREATMENT	0,5677	ns	

Fig. 4A

APX DUKE			
Source of Variation	P value	P value summary	
Interaction	0,0196	*	
RIPENING STAGE	0,5861	ns	
TREATMENT	<0.0001	****	

APX BRIGITTA			
Source of Variation	P value	P value summary	
Interaction	0,0039	**	
RIPENING STAGE	<0.0001	****	
TREATMENT	0,0001	***	

Fig. 4B

MDHAR DUKE			
Source of Variation	P value	P value summary	
Interaction	0,2599	ns	
RIPENING STAGE	<0.0001	****	
TREATMENT	<0.0001	****	

MDHAR BRIGITTA			
Source of Variation	P value	P value summary	
Interaction	0,6106	ns	
RIPENING STAGE	0,0002	***	
TREATMENT	0,1242	ns	

Fig. 4C

DHAR DUKE		
Source of Variation	P value	P value summary
Interaction	0,0126	*
RIPENING STAGE	<0.0001	****
TREATMENT	0,1245	ns

DHAR BRIGITTA		
Source of Variation	P value	P value summary
Interaction	0,8102	ns
RIPENING STAGE	0,001	**
TREATMENT	0,0014	**

Fig. 4D

GR DUKE		
Source of Variation	P value	P value summary
Interaction	<0.0001	****
RIPENING STAGE	<0.0001	****
TREATMENT	<0.0001	****

GR BRIGITTA		
Source of Variation	P value	P value summary
Interaction	0,8356	ns
RIPENING STAGE	0,0384	*
TREATMENT	0,1354	ns

Fig. 5A

DUKE PHENOLICS		
Source of Variation	P value	P value summary
Interaction	0,0835	ns
RIPENING STAGE	<0.0001	****
TREATMENT	<0.0001	****

BRIGITTA PHENOLICS		
Source of Variation	P value	P value summary
Interaction	0,9679	ns
RIPENING STAGE	0,0038	**
TREATMENT	<0.0001	****

Fig. 5B

DUKE ANTHOCYANINS		
Source of Variation	P value	P value summary
Interaction	0,0049	**
RIPENING STAGE	<0.0001	****
TREATMENT	<0.0001	****

BRIGITTA ANTHOCYANINS		
Source of Variation	P value	P value summary
Interaction	0,0012	**
RIPENING STAGE	<0.0001	****
TREATMENT	<0.0001	****

Fig. 5C

DUKE FLAVONOIDS		
Source of Variation	P value	P value summary
Interaction	0,8866	ns
RIPENING STAGE	<0.0001	****
TREATMENT	<0.0001	****

BRIGITTA FLAVONOIDS		
Source of Variation	P value	P value summary
Interaction	0,5511	ns
RIPENING STAGE	<0.0001	****
TREATMENT	<0.0001	****

Fig. 6A left (µg/g)

DELPH DUKE		
Source of Variation	P value	P value summary
Interaction	<0.0001	****
RIPENING STAGE	<0.0001	****
TREATMENT	<0.0001	****

CYAN DUKE		
Source of Variation	P value	P value summary
Interaction	<0.0001	****
RIPENING STAGE	<0.0001	****
TREATMENT	<0.0001	****

PETUN DUKE		
Source of Variation	P value	P value summary
Interaction	<0.0001	****
RIPENING STAGE	<0.0001	****
TREATMENT	<0.0001	****

PEON DUKE		
Source of Variation	P value	P value summary
Interaction	<0.0001	****
RIPENING STAGE	<0.0001	****
TREATMENT	<0.0001	****

MALV DUKE		
Source of Variation	P value	P value summary
Interaction	<0.0001	****
RIPENING STAGE	<0.0001	****
TREATMENT	<0.0001	****

Fig. 6A right (%)

DELPH DUKE		
Source of Variation	P value	P value summary
Interaction	<0.0001	****
RIPENING STAGE	<0.0001	****
TREATMENT	0,0552	ns

CYAN DUKE		
Source of Variation	P value	P value summary
Interaction	0,0004	***
RIPENING STAGE	<0.0001	****
TREATMENT	0,8409	ns

PETUN DUKE		
Source of Variation	P value	P value summary
Interaction	<0.0001	****
RIPENING STAGE	<0.0001	****
TREATMENT	<0.0001	****

PEON DUKE		
Source of Variation	P value	P value summary
Interaction	0,47	ns
RIPENING STAGE	<0.0001	****
TREATMENT	0,1925	ns

MALV DUKE		
Source of Variation	P value	P value summary
Interaction	<0.0001	****
RIPENING STAGE	<0.0001	****
TREATMENT	0,3876	ns

Fig. 6B left (µg/g)

DELPH BRIGITTA		
Source of Variation	P value	P value summary
Interaction	<0.0001	****
RIPENING STAGE	<0.0001	****
TREATMENT	<0.0001	****

CYAN BRIGITTA		
Source of Variation	P value	P value summary
Interaction	<0.0001	****
RIPENING STAGE	<0.0001	****
TREATMENT	<0.0001	****

PETUN BRIGITTA		
Source of Variation	P value	P value summary
Interaction	<0.0001	****
RIPENING STAGE	<0.0001	****
TREATMENT	<0.0001	****

PEON BRIGITTA		
Source of Variation	P value	P value summary
Interaction	0,0003	***
RIPENING STAGE	<0.0001	****
TREATMENT	<0.0001	****

MALV BRIGITTA		
Source of Variation	P value	P value summary
Interaction	0,0021	**
RIPENING STAGE	<0.0001	****
TREATMENT	0,0012	**

Fig. 6B right (%)

DELPH BRIGITTA		
Source of Variation	P value	P value summary
Interaction	<0.0001	****
RIPENING STAGE	0,0002	***
TREATMENT	0,511	ns

CYAN BRIGITTA		
Source of Variation	P value	P value summary
Interaction	0,7163	ns
RIPENING STAGE	<0.0001	****
TREATMENT	0,1687	ns

PETUN BRIGITTA		
Source of Variation	P value	P value summary
Interaction	0,0851	ns
RIPENING STAGE	<0.0001	****
TREATMENT	0,0001	***

PEON BRIGITTA		
Source of Variation	P value	P value summary
Interaction	0,9676	ns
RIPENING STAGE	<0.0001	****
TREATMENT	0,0018	**

MALV BRIGITTA		
Source of Variation	P value	P value summary
Interaction	<0.0001	****
RIPENING STAGE	<0.0001	****
TREATMENT	0,0576	ns

Fig. 7A

DI-SUBST DUKE		
Source of Variatior	P value	P value summary
Interaction	0,0013	**
RIPENING STAGE	<0.0001	****
TREATMENT	0,4507	ns

TRI-SUBST DUKE		
Source of Variatior	P value	P value summary
Interaction	0,0013	**
RIPENING STAGE	<0.0001	****
TREATMENT	0,4505	ns

METHOX DUKE		
Source of Variatior	P value	P value summary
Interaction	<0.0001	****
RIPENING STAGE	<0.0001	****
TREATMENT	0,1637	ns

Fig. 7B

DI-SUBST BRIGITTA		
Source of Variation	P value	P value summary
Interaction	0,7463	ns
RIPENING STAGE	<0.0001	****
TREATMENT	0,4336	ns

TRI-SUBST BRIGITTA		
Source of Variation	P value	P value summary
Interaction	0,6465	ns
RIPENING STAGE	<0.0001	****
TREATMENT	0,9967	ns

METHOX BRIGITTA		
Source of Variation	P value	P value summary
Interaction	<0.0001	****
RIPENING STAGE	<0.0001	****
TREATMENT	0,4185	ns
